# Artificial intelligence for early stroke diagnosis in acute vestibular syndrome

**DOI:** 10.3389/fneur.2022.919777

**Published:** 2022-09-08

**Authors:** Athanasia Korda, Wilhelm Wimmer, Thomas Wyss, Efterpi Michailidou, Ewa Zamaro, Franca Wagner, Marco D. Caversaccio, Georgios Mantokoudis

**Affiliations:** ^1^Department of Otorhinolaryngology, Head and Neck Surgery, Inselspital, University Hospital Bern and University of Bern, Bern, Switzerland; ^2^Hearing Research Laboratory, ARTORG Center, University of Bern, Bern, Switzerland; ^3^University Institute of Diagnostic and Interventional Neuroradiology, Inselspital, University Hospital Bern and University of Bern, Bern, Switzerland

**Keywords:** vertigo, artificial intelligence, video head impulse test, stroke diagnosis, emergency department

## Abstract

**Objective:**

Measuring the Vestibular-Ocular-Reflex (VOR) gains with the video head impulse test (vHIT) allows for accurate discrimination between peripheral and central causes of acute vestibular syndrome (AVS). In this study, we sought to investigate whether the accuracy of artificial intelligence (AI) based vestibular stroke classification applied in unprocessed vHIT data is comparable to VOR gain classification.

**Methods:**

We performed a prospective study from July 2015 until April 2020 on all patients presenting at the emergency department (ED) with signs of an AVS. The patients underwent vHIT followed by a delayed MRI, which served as a gold standard for stroke confirmation. The MRI ground truth labels were then applied to train a recurrent neural network (long short-term memory architecture) that used eye- and head velocity time series extracted from the vHIT examinations.

**Results:**

We assessed 57 AVS patients, 39 acute unilateral vestibulopathy patients (AUVP) and 18 stroke patients. The overall sensitivity, specificity and accuracy for detecting stroke with a VOR gain cut-off of 0.57 was 88.8, 92.3, and 91.2%, respectively. The trained neural network was able to classify strokes with a sensitivity of 87.7%, a specificity of 88.4%, and an accuracy of 87.9% based on the unprocessed vHIT data. The accuracy of these two methods was not significantly different (*p* = 0.09).

**Conclusion:**

AI can accurately diagnose a vestibular stroke by using unprocessed vHIT time series. The quantification of eye- and head movements with the use of machine learning and AI can serve in the future for an automated diagnosis in ED patients with acute dizziness. The application of different neural network architectures can potentially further improve performance and enable direct inference from raw video recordings.

## Introduction

Strokes presenting with symptoms of dizziness or vertigo often mimic benign inner ear diseases, which can lead to misdiagnosis by physicians (1). Failure to rapidly diagnose and promptly treat such strokes often results in disability or death (2). Strokes occur in up to 8% of dizzy patients presenting in the emergency department (ED) (3) and any support tool reducing stroke misdiagnosis is very important.

Currently, the most widely accepted triage tool for stroke detection in dizzy patients in the ED is the “HINTS” eye movement examination (4). “HINTS” is used as an acronym for the head impulse test, nystagmus test and test of skew. Such a clinical test can be applied in a timely and efficient manner at the bedside. However, the correct application and test assessment needs expertise, which is not always readily available. Even experts struggle with the assessment of head impulses when hidden (covert) corrective saccades and spontaneous nystagmus occur (5). In comparison to a clinical assessment, videooculography (VOG) devices enables a quantification of eye- and head movements at the bedside, which can improve the accuracy of HINTS (6, 7). The Vestibular-Ocular-Reflex (VOR) gain by video head impulse (vHIT), especially, has already been successfully used to differentiate between central and peripheral causes in patients with an acute vestibular syndrome (AVS) (6, 8).

These VOG devices are easy to use (9) and they can serve in the near future with telemedicine (10) and machine intelligence in a remoted setting such as smaller community hospitals lacking onsite experts, or in pandemic times as a diagnostic tool for acute dizziness (11, 12). VOG could potentially support physicians in the ED analog to an Eye ECG (13). Artificial intelligence (AI) has been suggested to improve stroke diagnosis in EDs, by implementing machine learning-enabled clinical decision support systems (14, 15). A concrete application of deep learning to vestibular disorder classification using videonystagmography was presented by Ben Slama et al. (16). The advantage of AI applied on raw VOG data for its assessment is the holistic approach on unprocessed head impulse test data compared to partial assessments such as VOR gain at one single time point or saccade latencies. Current analysis of vHIT data depend on the parameters assessed and the associated calculation methods (17).

In this study, we tried to test automated AI stroke classification based on vHIT time series and to compare whether the accuracy of the AI-based method is comparable to VOR gain based stroke classification.

## Materials and methods

### Study design and patient characteristics

In this prospective, cross-sectional study, data were collected in the ED during office hours between 07/2015 and 04/2020, which was part of a larger study (DETECT–Dizziness Evaluation Tool for Emergent Clinical Triage). The local ethics committee (Kant. Ethikkommission Bern) approved this study (KEK # 047/14). We included patients with AVS who had a continuous dizziness, associated with nausea or vomiting, head-motion intolerance, new gait or balance disturbance, and nystagmus. We excluded patients younger than 18 years, if symptoms abated after 24 h, or if the index ED visit was >72 h after symptom onset. Patients with previous eye movement or vestibular disorders were also excluded. All enrolled patients gave written consent.

### vHIT measurements

A subset of VOR gain data presented here have been published elsewhere (6, 18–21). A neurootologist with 2 years' experience in the field, performed physical examination, Caloric Testing, and vHIT testing in all enrolled patients. vHIT was performed using the EyeSeeCam (EyeSeeTec GmbH) (22) and by applying fast passive horizontal head movements (high frequency, 10–20° head excursion in 100–300 milliseconds corresponding to a 1,000–6,000°/sec^2^ acceleration) in room light during visual target fixation at more than 1m distance. We assessed only data from valid vHIT marked by the device following data quality criteria such as peak head velocity exceeding 70°/s within the first 150 milliseconds with a head exceeding 1,000°/sec^2^. Head impulses were excluded if the eyes or head were moving (>20°/s) before the onset of the head impulse or if the direction of the head impulse was not in the horizontal plane (i.e., within ±45°). Outliers regarding peak head velocity (1.5-fold interquartile range) were rejected (23). Two neurootologists (GM, AK) in a consensus meeting reviewed all vHITs for data quality and artifacts. Only clean data with non-disruptive artifacts were included based on a predefined classification (24).

### Patient labeling and stroke diagnosis

All patients received an acute MR brain scan either within 48 h in the ED or a second, delayed MRI (3–10 days after symptoms onset), if there was no acute MRI indicated based on clinical grounds or if the first acute MRI was non-diagnostic. The delayed MRI served as a gold standard for stroke detection. A blinded experienced board-certified senior consultant in neuroradiology re-assessed all MRIs. Patients with a negative MRI and a pathological caloric test were classified as acute unilateral vestibulopathy (AUVP) / vestibular neuritis.

### VOR gain based stroke classification

VOR gain values were derived from eye velocity divided by head velocity at 60 ms after HIT onset. We calculated a best discrimination cut-off for stroke by applying a receiver operating characteristics curve (ROC). We did not use saccade analysis, since the currently used VOG software did not offer an automated feature for saccade analysis.

### AI-based stroke classification of VOG

**Figure 1:**
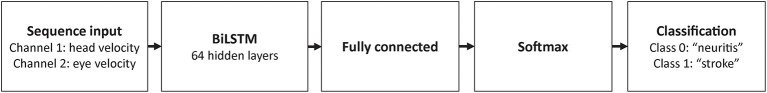
Diagram of the recurrent neural network architecture used for the classification of VOG times series. A bidirectional long short-term memory (BiLSTM) model was used to enable context awareness between past and following sequences in a given time series of a patient.

All data were evaluated in a time course between “start of head impulse” (which was defined as the point 250 ms before the maximal head velocity) and 700 ms after head movement stopped. Then all head impulses for both horizontal directions (right, left) of a single patient were concatenated into time series with two channels (channel 1: head velocity, channel 2: eye velocity). For classification, a neural network using a long short-term memory architecture with 64 hidden layers was trained with a batch size of 512 samples for 256 epochs (Figure 1). The neural net was implemented using MATLAB (Version R2020b, Mathworks, Inc., Natick, US). The data was split in a 70% training set (*N* = 40 patients) and a 30% test data set (*N* = 17 patients). The assignment of patients for the training and validation data set were randomly shuffled before each training epoch of the neural network to avoid overfitting to the training data set. To account for different time series lengths for individual patients, all input data were segmented into data-streams of 512 samples, typically covering 3 consecutive vHITs. One of our goals was to reduce the preprocessing of the data to a minimum. Therefore, we wanted the neural network to be able to process vHIT time sequences with different lengths (depending on the number of tests performed and the recording duration). Since our neural network requires input streams with constant length, we needed to find a suitable length for the data streams to avoid extensive padding and truncating. In our data, most sequences had about 4,100 samples, the shortest had 1,200 samples, and the longest consisted of 9,266 samples. To cover this range, we chose a stream length of 512 samples (practical sample size as a power of 2), which approximately corresponds to 3 vHITs. We also tried other sequence lengths, but found that a data stream length of 512 samples and a mini-batch size of 512 samples worked well. Data balancing was performed to avoid a biased training outcome of the network toward the more frequent “no stroke” cases (AUVP) by duplicating sequences (over-sampling) of the stroke data set to result in an equal amount of AUVP and stroke sequences. In total, this resulted in 535 data-streams for training and 233 data-streams for testing. No filtering of the time series was performed. All data streams were standardized by subtracting the overall mean value of the head and eye velocities and dividing by the standard deviation.

### Statistical analysis

Descriptive statistics were reported using SPSS statistical software (IBM SPSS Statistics for Windows, Version 25.0. Armonk, NY: IBM Corp.). We used a binary logistic regression to evaluate stroke predictors derived from VOR gains and AI-Scores. We calculated a receiver characteristics curve (ROC) with its corresponding sensitivity, specificity, and accuracy for each test. Best cut-off points based on Youden's J. The two ROC curves were compared using the method of DeLong et al. (25).

## Results

We analyzed data from 57 patients aged between 30 and 78 years (average 55 years) with a diagnosis of stroke or AUVP and valid vHIT measurements. Gold standard classification assigned (39 with AUVP and 18 with stroke).

### VOR-gain based stroke classification of vHIT

We found odds ratio of 3.3 with a significant increase of stroke probability for each VOR gain increment of 1.194 (*p* < 0.001, CI 1.785–6.106) (see Table 1). The overall sensitivity and specificity for detecting a stroke with a VOR gain cut-off of 0.57 was 88.8 and 92.3% respectively and thus the accuracy was 91.2% (Table 2 and **Figure 3**).

**Table 1:** Logistic regression and predictive variables.

**Test variable**	**Regression coefficient**	**Standard error**	**Wald**	**df**	***p*-Value**	**Odds ratio**	**95% CI**	
							**Lower limit**	**Upper limit**
VOR gain	1.194	0.314	14.500	1	<0.001	3.302	1.785	6.106
AI-score	0.422	0.046	84.698	1	<0.001	1.525	1.394	1.669

CI 95% confidence intervals, df degree of freedom.

**Table 2:** ROC curve.

	**VOR gain**	**AI-score**
Area under the curve	0.95	0.88
Std-error	0.03	0.23
*p*-Value	0.00	0.00
95% Lower limit	0.89	0.83
95% Upper limit	1.00	0.93
Positive, if smaller or equal^*^	0.57	0.46
Sensitivity	0.88	0.87
Specificity	0.92	0.88
Accuracy	0.91	0.87
Negative predictive value	0.95	0.80
Positive predictive value	0.84	0.92
Positive likelihood ratio	11	7.25
Negative likelihood ratio	0.13	0.14

* cut-off.

### AI-based stroke classification of vHIT

**Figure 2:**
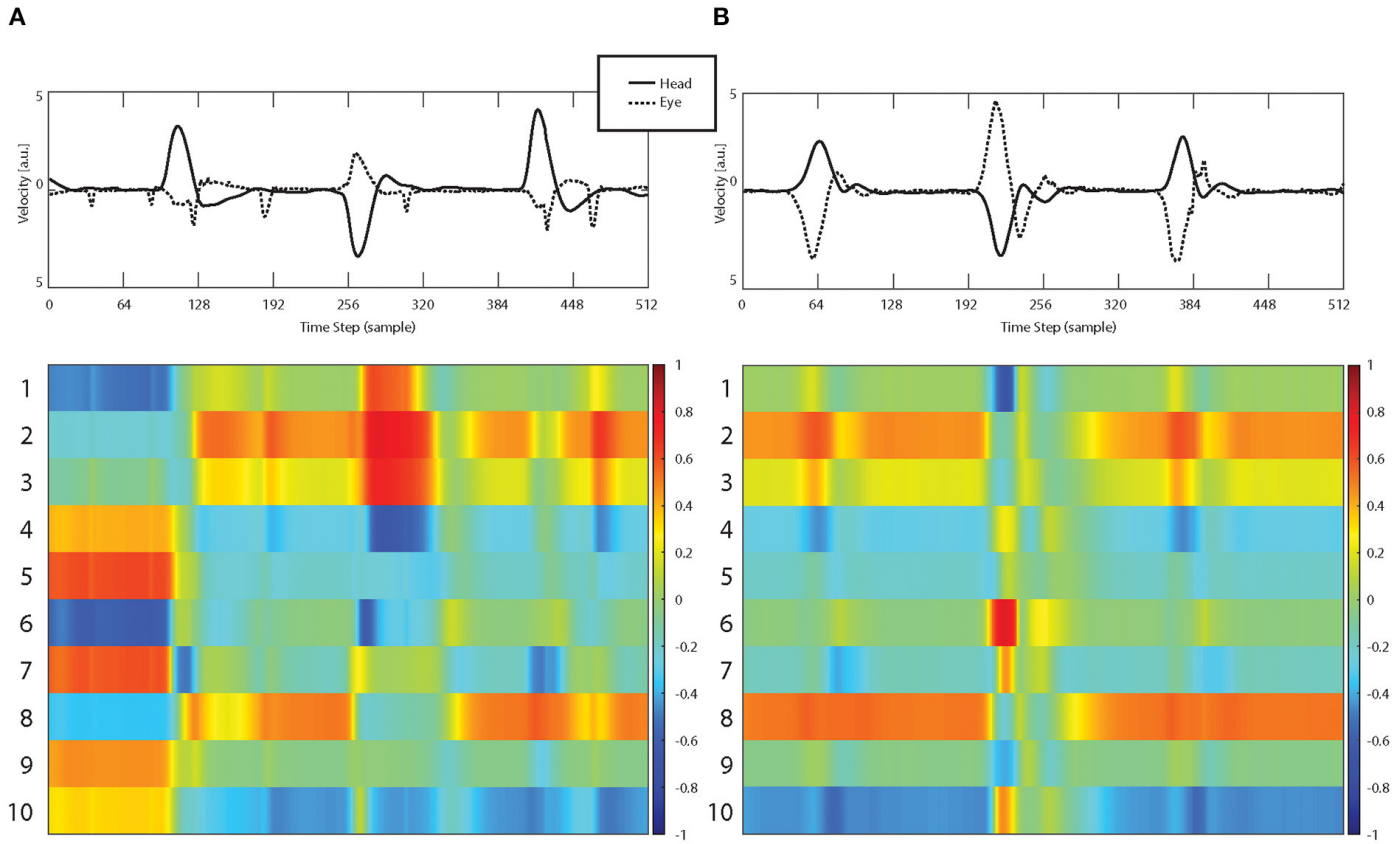
Examples of vHIT input streams (top row, raw data including artifacts) consisting of eye velocity (dashed curve) and head velocity (continuous curve) time series and corresponding activation patterns of the first 10 hidden LSTM layers for a patient with AUVP **(A)** and a stroke patient **(B)**.

**Figure 3:**
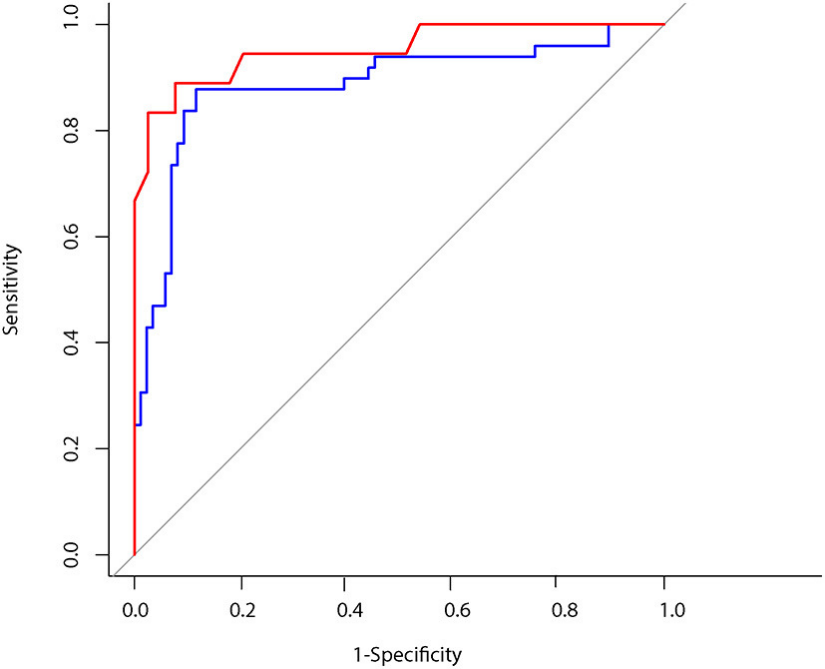
Blue line: ROC CURVE using artificial intelligence for head impulse test interpretation AUC: 0.88. Red line: ROC CURVE using VOR gain to predict a vestibular stroke AUC: 0.95.

Table 1 shows the odds ratio of 1.52 with a significant increase of stroke probability for each AI score increment of 0.422 (*p* < 0.001, CI 1.394–1.669). The obtained network was able to classify strokes with an accuracy of 87.9% with a sensitivity of 87.7% and specificity of 88.4% (Table 2 and **Figure 3**). Example of the neural network activation patterns for a data-stream of an AUVP and a stroke patient are shown in Figure 2. Artifacts such as goggles slippage or head overshoot at the HIT end (Figure 2) were occurring randomly (random noise/variation) (17) with no systematic bias. There was no statistical difference between the two ROC curves (*p* = 0.92) and thus, there was no inferiority regarding AI classification (Figure 3).

## Discussion

Our study showed that AI-based classification of unprocessed vHIT time series has a high accuracy und is as accurate as the VOR gain classification for differentiation between vestibular strokes and peripheral AVS.

### Machine learning and the head impulse test

Our analysis showed encouraging results using a recurrent neural network architecture (long short-term memory) for the binary classification task (“stroke” vs. “no-stroke”) of VOG time series as exported by the diagnostic software. The input time series were eye (from one eye only) and head velocities, taken from head impulses.

VOR gain using a vHIT device can be calculated by various methods (17). We used the velocity gain at 60 ms in order to avoid any bias from covered saccades or spontaneous nystagmus. Gain calculation might be more susceptible to artifacts and wrong eye calibrations resulting in wrong gain estimations compared to AI, which considers the whole velocity profile data. Therefore, it is mandatory to inspect visually the velocity profile of slow phase VOR which needs to be bell-shaped and not contaminated with artifacts. Such manual assessment needs expertise by the examiner.

With the advent of machine learning, these steps can be combined into a single machine-learning instance that is trained to directly classify eye movement video recordings (26). However, there are two steps that need to occur prior to trying to classify any new recording. First, a large dataset must be collected and labeled by a set of experts. This labeling must correspond with the indented classification to be performed by the machine. For example, a recording in a dataset could be labeled as stroke or no stroke according to neuroimaging results. Then, the machine is trained with this dataset and becomes ready to classify new recordings. Machine learning may increase its diagnostic accuracy by combining results and features obtained from multiple tests like nystagmus test and test of skew (14), or can also be used to only replace an individual step or group of steps in the classical analysis pipeline.

While other studies used different data sets to apply AI on vestibular disorders (14, 27, 28), we chose to try AI in vHIT data because HIT has been previously considered the most important component of HINTS with a 18-fold stroke probability in AVS patients with a bilateral normal HIT (29). Accuracy of AI in stroke detection depends not only on the quality of disease labeling but also on the quality of the collected data. An expert can improve data quality of each performed impulse by encouraging subjects to keep their eyes open and by avoiding any physical contact between the examiners hand and the goggles. Applying specific recording techniques and avoiding some known pitfalls during eye- and head tracking minimize the risk of artifacts (9, 24, 30). Moreover, data from all impulses are averaged, further reducing the effect of noise or artifacts in single impulse (24).

Our results of VOR gain accuracy are similar to older studies (8). The known dissociation between caloric test results and vHIT might affect the overall specificity of this test (6). A recent study showed a slight worsening of accuracy of AI for stroke classification based on HINTS data (14). This discrepancy may be explained by the fact that we used different data forms. We used unprocessed raw data (including artifacts), whereas they used only pre-processed/calculated VOR gain values for AI. A single VOR gain number does not reflect the whole dynamic of a head impulse and informative data such as corrective saccades are completely ignored. Our approach, however, included the whole vHIT trace including the complete slow phase VOR (ascending, peak and descending velocity profile) and the fast phase responses (compensatory saccades) for a period of 700 ms. This more holistic approach explains the improved accuracy of AI for stroke classification.

### Strength and limitations

To our knowledge, this is the first study, which used AI in raw vHIT time series for stroke classification in AVS. The biggest limitation of our study is the small sample size used to train the neural net. For this reason, although we obtained promising results, our study should be considered as exploratory. More training data from multicenter prospective studies may improve the performance, as data set size is usually a limiting factor in machine-learning studies (31). The long short-time memory architecture is a commonly used model for the classification of time series. We can envision the use of other network architectures, e.g., used for image segmentation and classification tasks to directly utilize raw video recordings as predictive variables. Moreover, additional data, such as gyroscope recordings can be included for improved robustness. In addition, we did not analyze other tests such as nystagmus and test of skew. We expect that the combination of several tests (which is reflected in the three steps “HINTS” exam), would further improve AI sensitivity (32). AVS patients suffer from imbalance and gait disturbance. Additional tests, such the assessment of stance and gait, can already be assessed automatically by the application of machine learning (14, 33) and might be added in future triage protocols.

The application of head impulse data for stroke classification is restricted only for AVS patients and should not be applied to every acute vertigo patient (30). This fact means that AI based ED triage with the head impulse test can only be applied on selected patients with true AVS and is not generalizable to all dizzy ED patients. Patients with other causes of vertigo such as benign paroxysmal positional vertigo (BPPV) should be evaluated by positional tests either on site or remotely by the application of telehealth programs (34). Other modern machine learning methods can be successfully applied on patients with recurrent vertigo (spontaneous episodic vertigo syndromes) such as Menière's disease and vestibular migraine (35). It might also be used for the triage of common vestibular disorders however, (36) current classification accuracy is still low.

### Clinical implications

The prospective collection of big data is the prerequisite for a future successful implementation of AI in clinical decision support systems (37–39).

An application of AI on big dizziness data repositories in the future can lead to a development of an automated interpretation of VOG results or automated early stroke detection in at risk dizzy patients. Clinical decision support systems are highly recommended for the assessment of the vHIT or “HINTS,” since computer algorithms assess more than single VOR gain values or catch-up saccade frequency. Bedside clinical HIT tests, however, rely exclusively on the presence of catch-up saccades and need further expertise, which is not readily available in the ED. We recommend, therefore, future multicentric observational studies with systematic quantitative recordings of eye- and head movements combined with telemedicine services on every dizzy patient. Such big data approach has the potential for an automated VOG triage as a point-of-care decision support tool. We, therefore, believe that a more holistic approach offered by AI could not only pave the way for a widespread use of vHIT in EDs but could also substantially improve the objective assessment of vHIT at the bedside.

## Conclusion

AI can accurately diagnose a vestibular stroke by using only vHIT unprocessed data in patients with AVS. Automated vHIT assessment for stroke prediction was not inferior to the current approach assessing a single VOR gain value. However, the algorithm might be further improved by larger training data sets and the implementation of additional tests collected with VOG at the bedside. The quantification of eye- and head movements with the use of machine learning and AI is a promising future tool for an automated diagnosis in ED patients with acute dizziness.

## Data availability statement

The original contributions presented in the study are included in the article/supplementary material, further inquiries can be directed to the corresponding author.

## Ethics statement

The study was approved by the Local Ethics Committee (KEK # 047/14). The patients/participants provided their written informed consent to participate in this study.

## Author contributions

AK: investigation, data curation, and writing—original draft. WW: conceptualization, methodology, formal analysis, and writing—review and editing. TW and EM: data curation. EZ and FW: investigation. MC: supervision and project administration. GM: conceptualization, formal analysis, writing—review and editing, project administration, and funding acquisition. All authors contributed to the article and approved the submitted version.

## Funding

This study was supported by the Swiss National Science Foundation #320030_173081.

## Conflict of interest

The authors declare that the research was conducted in the absence of any commercial or financial relationships that could be construed as a potential conflict of interest.

## Publisher's note

All claims expressed in this article are solely those of the authors and do not necessarily represent those of their affiliated organizations, or those of the publisher, the editors and the reviewers. Any product that may be evaluated in this article, or claim that may be made by its manufacturer, is not guaranteed or endorsed by the publisher.

## Abbreviations

AVS: acute vestibular syndrome
AUVP: acute unilateral vestibulopathy
HINTS: Head-Impulse-Nystagmus-Test-of-Skew
ED: emergency department.
